# Evaluating Research and Impact: A Bibliometric Analysis of Research by the NIH/NIAID HIV/AIDS Clinical Trials Networks

**DOI:** 10.1371/journal.pone.0017428

**Published:** 2011-03-04

**Authors:** Scott R. Rosas, Jonathan M. Kagan, Jeffrey T. Schouten, Perry A. Slack, William M. K. Trochim

**Affiliations:** 1 Concept Systems, Inc., Ithaca, New York, United States of America; 2 Division of Clinical Research, National Institute of Allergy and Infectious Diseases/National Institutes of Health, Bethesda, Maryland, United States of America; 3 HIV/AIDS Network Coordination Project, Fred Hutchinson Cancer Research Center, Seattle, Washington, United States of America; 4 Department of Policy Analysis and Management, Cornell University, Ithaca, New York, United States of America; Karolinska Institutet, Sweden

## Abstract

Evaluative bibliometrics uses advanced techniques to assess the impact of scholarly work in the context of other scientific work and usually compares the relative scientific contributions of research groups or institutions. Using publications from the National Institute of Allergy and Infectious Diseases (NIAID) HIV/AIDS extramural clinical trials networks, we assessed the presence, performance, and impact of papers published in 2006–2008. Through this approach, we sought to expand traditional bibliometric analyses beyond citation counts to include normative comparisons across journals and fields, visualization of co-authorship across the networks, and assess the inclusion of publications in reviews and syntheses. Specifically, we examined the research output of the networks in terms of the a) presence of papers in the scientific journal hierarchy ranked on the basis of journal influence measures, b) performance of publications on traditional bibliometric measures, and c) impact of publications in comparisons with similar publications worldwide, adjusted for journals and fields. We also examined collaboration and interdisciplinarity across the initiative, through network analysis and modeling of co-authorship patterns. Finally, we explored the uptake of network produced publications in research reviews and syntheses. Overall, the results suggest the networks are producing highly recognized work, engaging in extensive interdisciplinary collaborations, and having an impact across several areas of HIV-related science. The strengths and limitations of the approach for evaluation and monitoring research initiatives are discussed.

## Introduction

In 2006, the US National Institute of Allergy and Infectious Diseases (NIAID) restructured its system of extramural HIV/AIDS clinical trials networks prompted by changes in the infectious disease knowledge base, the unabated expansion and shifting demographics of the epidemic, the size and complexity of the enterprise, and fiscal considerations. The six networks are the AIDS Clinical Trials Group, HIV Prevention Trials Network, HIV Vaccine Trials Network, Microbicide Trials Network, International Maternal Pediatric and Adolescent Trials Network, and the International Network for Strategic Initiatives in Global HIV Trials. Six scientific priority areas were identified [1] and, an extensive participatory evaluation planning effort was undertaken [2]. During this process, system stakeholders identified several key areas for evaluation, with highest priority given to the assessment of the networks' scientific agendas and biomedical research objectives. More specifically, the extent to which network research is highly influential, addressing the highest priority research questions, making significant progress in planned research, and informing standards of care or clinical guidelines were viewed as key evaluation questions. This report presents results from bibliometric analyses based on the networks' research publication record for the period 2006–2008 and considers the strengths and drawbacks of this approach in assessing the impact and performance of scientific research output for this (and potentially other) clinical trials programs.

### Bibliometric studies of HIV research

Previous bibliometric studies describing publication trends and patterns in HIV research provided a base of understanding for the ways bibliometrics might be applied to the evaluation of the scientific output from the NIAID HIV/AIDS clinical trials networks. A co-citation analysis of HIV research completed in the early years of the epidemic traced the expansion of the field and shifts in study foci [3]. Further, bibliometric studies of the rapidly transforming HIV knowledge base captured the presence of new scientific terminology and the specialization of journals as the field progressed [4]–[6]. The emergence of the study of HIV as an interdisciplinary field of research, coupled with the advancement of bibliometric methods over the past 25 years, has enabled researchers to better assess collaboration patterns, geographic distribution, and expansion of subject areas and content of the science. Reflecting the global scientific effort to address the epidemic, an array of bibliometric studies of research output in international settings [7]–[14], as well as work documenting the productivity and collaboration between different world regions have been published [15].

While these earlier bibliometric studies of HIV research have described the distribution and variation in the scientific output over time, the use of bibliometric methods to determine the baseline level of performance and impact of research output from a large system of HIV clinical trials research has not been carried out previously. To that end, our purpose was to assess the presence, performance, and impact of scientific publications by the NIAID HIV/AIDS clinical trials networks since the 2006 reorganization through a comprehensive bibliometric evaluation that included normative comparisons, collaborative network analyses, and inclusion in research syntheses.

## Methods

### Evaluation of scientific output

The evaluation of scientific work is among the key driving forces behind modern scientific advancements. Evaluative bibliometrics seeks to assess the impact of scientific output in the context of other published science and usually compares the relative scientific contributions of research groups or institutions. Several evaluations of large, publicly-funded research programs have conducted bibliometric analysis as one component of a comprehensive and integrated evaluation approach designed to assess processes and outcomes of scientific initiatives using quantitative indicators that enable aggregation of output [16]–[18]. While the application of bibliometrics to aggregate and analyze the scientific output across research units and centers has increased, the use of such data for evaluation within a specific research enterprise requires a clear purpose, context, and understanding of the limitations.

Bibliometrics involves the quantitative assessment of the occurrence of certain events in the scientific literature, as opposed to the analysis and interpretation of the literature's content. The use of bibliometrics relies on the very structured nature and expectations of the refereed scientific literature. The primary assumption supporting the use of bibliometrics is that exchange and recognition of research results is desired and is one of the key driving forces in the advancement of science [19]. Citations symbolize the association of scientific ideas, and the references which authors cite in their papers make explicit the link between their current research and prior work in the scientific literature archive. Therefore, the analysis of publication data can help quantify the performance and impact of a given set of publications produced by an entity as it relates to the exchange and dissemination of results.

Results from bibliometric analyses can be a critically important source of objective information about the quantity and quality of scientific work. Three basic tenets underlying advanced bibliometric analyses have been emphasized in the literature: a) activity measurement; b) impact measurement, and c) linkage measurement [20]. Of these three tenets, impact measurement has been the most tenuous, conceptually and methodologically. Because of the well-known limitations of bibliometric data, claims of impact have been extensively debated. However, as the use of bibliometric methods to evaluate scientific outcomes has increased in recent years, so too has the attention to the fundamental concepts, language, techniques, and implications for evaluation. Furthermore, new network analysis and visualization techniques are being used more frequently to model complex relationships of scientific output.

In our assessment of the performance and impact of the research output from the NIAID HIV/AIDS clinical trials networks, we followed the advanced bibliometric methods approach for monitoring and evaluating research units outlined by the Center for Science and Technology Studies at Leiden University [21]–[23]. One strand of this approach focuses on research performance evaluation of a well-defined entity and is particularly useful in new problem-oriented interdisciplinary fields. The other focuses on defining an emerging field of interdisciplinary research where the boundaries are not clearly established. The evaluation of the scientific output of the NIAID HIV/AIDS clinical trials networks presented the opportunity to employ techniques from both strands to describe the impact of the current research in the context of an expanding interdisciplinary scientific landscape.

In evaluating the NIAID HIV/AIDS clinical trials networks as an entity, we examined the research output in terms of its presence in the scientific journal hierarchy, performance of publications on widely accepted bibliometric measures, and impact of the research relative to other papers published worldwide. Through the creation of a research profile of publication performance and impact across journal fields [21] we sought to identify specific fields in which publications from the networks were exhibiting the greatest performance and impact in the relevant scientific literature. Additionally, we isolated a set of highly performing papers to analyze their presence, performance, and impact across a variety of fields and journals. Lastly, in order to look at interdisciplinarity, we examined international cooperation and co-authorship patterns, as well as the inclusion of papers in reviews and information syntheses.

### Data source and sample

Each of the six clinical research networks (i.e., AIDS Clinical Trials Group, HIV Prevention Trials Network, HIV Vaccine Trials Network, Microbicide Trials Network, International Maternal Pediatric and Adolescent Trials Network, and the International Network for Strategic Initiatives in Global HIV Trials) was asked to submit a list of its network supported publications appearing in peer-reviewed journals between 2006 and 2008. All networks complied and provided a combined electronic bibliography of 450 publications for the specified period. After verifying the full bibliographic entry for each article in PubMed, the final set of 450 articles was searched via the ISI-Thomson Web of Science (WoS) platform. WoS is the leading source for bibliometric citation databases and includes the Science Citation Index-Expanded and the Social Science Citation Index databases.

For retrieving journal specific citation metrics, the ISI Journal Citation Report (JCR) database was used. The JCR Science Edition indexes 7,350 leading peer-reviewed journals in 171 different subject categories and more than 2,242 leading peer-reviewed journals in 55 subject categories in its Social Science Edition. Both editions were used to retrieve field specific journal rankings. Approximately 25 million citations are processed annually for inclusion in the JCR database making it the most robust journal citation database available. We used these to benchmark and rank journals containing the NIAID HIV/AIDS clinical trials networks papers.

The initial extraction of citation data for the 450 articles yielded 426 matches, based on correspondence between indexed journals in the WoS databases and those found in the bibliography submitted by the networks. The 24 unmatched papers were published in journals that were not indexed in the scientific publication databases because they did not meet multi-component review criteria for peer-review quality or coverage. Two journals publishing five network articles, (*AIDS Research and Therapy* and *PLoS One*) had been evaluated in the past but not been accepted for indexing at the time of extraction due to the absence of a uniformly applied, formal peer review. Several other journals on the excluded list, including *Research Practitioner* (6 network articles) were not peer review journals, but as in the case of *Research Practitioner*, “edited with the highest editorial standards”. These journals were not indexed in either PubMed or MEDLINE as well. Although the WoS databases do not include all journals or capture every published article across fields, it is currently the best set of information available to examine the extent to which network publications are present in the overall body of HIV/AIDS literature.

The citation data from the 426 source articles referenced above were extracted and imported into a Microsoft Access database that allowed computation and analysis of citation information. Seven articles included in the extracted data set fell outside the 2006–2008 time period due to differences in the publication year recorded in the WoS databases. These papers were subsequently excluded from the bibliometric analyses. Thus, a final set of 419 publications met the timeframe requirements and had the citation data needed for the analyses described in this report.

## Results

### Descriptive findings

The 419 papers included in the analysis were published in 114 journals indexed by Thomson-ISI within the JCR database. Approximately 75% of the network papers were cited at least once. Overall, the 419 network papers were cited 2,582 times within 1,996 papers published in 549 indexed journals. Publications from the networks averaged 6.16 citations per paper with a median cite of 2. The number of citations ranged from 0 to 170. Over the three year period, 124 papers (29.5%) were uncited, after adjusting for self-citations. Self-citations refer to citations by any authors identical to those found on the cited paper. Because higher author self-citation rates can inflate the number of citations, the author self-citation rate was calculated for this set of papers to determine if the self-citation rate was substantial. Since many articles had multiple authors, and not all authors were researchers from the NIAID HIV/AIDS clinical trials networks, it was possible that self-citation included citation of an article by a non-network co-author. Based on the total number of citations for all 419 papers and the number of self-cites among all authors named for each publication (528), we found a self-cite rate of slightly more than 20% (20.4%). Studies have shown that authors working in research-based disciplines tend to cite themselves between 10% and 30%, depending upon the field of study [23]–[25]. Thus, the self-cite rate for the papers from the NIAID HIV/AIDS clinical trials networks is within the ‘normative’ range for author self-citation, despite the high level of co-authorship.

### Presence in journal hierarchy

Journals are not homogeneous outlets of science in terms of their audiences, visibility, significance, and readership. Across fields of research, great value is placed on journals with higher status and perceived levels of productivity and therefore attracts large international audiences from the scientific community. Journals with the propensity to draw a great deal of attention to the papers it publishes are held in high regard and widely recognized across international settings. Requirements for publishing in these journals are often stringent and review processes particularly demanding. Therefore, analyses that reveal where a set of papers reside in the journal hierarchy is important to evaluating the presence of a set of papers across scientific fields.

In our examination of the journals in which papers from the NIAID HIV/AIDS clinical trials networks were published, we found the 419 papers were published in 114 different journals, with the number of publications per journal ranging from 57 to 1. The average Journal Citation Report (JCR) impact factor for journals containing the set of 419 papers was 5.82 and values ranged from .16 to 52.59. Using the widely-available JCR impact factors as preliminary means for ranking journals we found 47 of the 114 journals (41.2%) publishing papers from the NIAID HIV/AIDS clinical trials networks were in the top 10% of *all* scientific journals indexed by Thomson-ISI. The average impact factor of this select group of 47 journals was 9.74, with nearly two-thirds of all of the network papers (n = 271; 65%) published in journals at this high level. This set of 271 papers published in the upper tier of scientific journals received 84.8% (2,189) of all citations attributed to publications from the NIAID HIV/AIDS clinical trials networks and the percentage of papers that were cited one or more times, including self-citations, was 81.2%. Thus, the majority of papers from the networks were published in highly productive and visible journals, based on a global ranking and therefore obtaining a substantial proportion of citations. Not surprisingly, more than one third of the papers from the NIAID HIV/AIDS networks (35.3%) were published in 3 specific journals: *AIDS* (JCR impact factor = 5.84), *Journal of Acquired Immune Deficiency Syndromes* (JCR impact factor = 4.41), *and Journal of Infectious Diseases* (JCR impact factor = 5.84) reflecting the trend of publication of AIDS research output in specialized journals over the past decade [3].

### Overall performance and impact of research output

To assess the performance of the set of 419 papers from the NIAID HIV/AIDS networks, we calculated the total number of citations for the set (*C*) and citations per paper (*CPP*) by year and publication type. Following procedures and terminology outlined by Center for Science and Technology Studies at Leiden University for assessing impact of a set of papers relative to those published worldwide, we accessed: a) the average total number of citations of a certain article type (abstract, article, review, note, etc.) published in a specific journal cumulatively by the most recent completed year (Journal Citation Score; JCS), and; b) the average number of citations for all articles that were published in a particular year, in all journals in a specific field (Field Citation Score; FCS) from the JCR database. The JCS represents the number of citations one would expect for a paper of the same type, published in the same journal, in the same year and serves as an international reference to compare relative impact of publications to those published in specific journals. The FCS represents the number of citations one would expect for a paper of the same type, published in all journals within a specific field in the same year, and serves as an international reference to compare relative impact of publications to those published in the group of journals that constitute a field. Next we summed all of the citations to the papers published by the NIAID HIV/AIDS networks for 2006–2008. We then summed all of the corresponding JCS values for each paper and calculated a journal normalized measured impact ratio (CPP/JCSm). Finally, we summed all of the corresponding FCS values for each paper and calculated a field normalized measured impact ratio (CPP/FCSm) [21]. In both cases, normalization of the citation values is completed at the group level after summing the world averages that correspond to the selected publications with respect to the publication type, age, and journal (for the JCS) or subject area (for the FCS) and then dividing it by the number of citations for the set of publications.

The results of the performance and impact of the NIAID HIV/AIDS clinical trials networks' publications is included in Table 1. A ratio of 1.0 indicates that the set of publications have met the expected number of citations based on the journals in which they were published. For the collection of 419 papers, the CPP/JCSm ratio was 1.12, indicating the papers from the NIAID HIV/AIDS clinical trials networks were cited 12% above average across the set of journals publishing papers produced by the networks. The CPP/FCSm ratio was 1.90, indicating the papers from the NIAID HIV/AIDS clinical trials networks were cited 90% above the worldwide average across relevant fields. In terms of evaluating the level of impact, generally accepted international impact standards for interpreting both the CPP/JCSm and CPP/FCSm ratios have been published [21]–[23]. Specifically, the levels are: far below average (indicator value<0.5); below average (indicator value 0.5–0.8); average (0.8–1.2); above average (1.2–1.5); and far above average (>1.5). Thus, the impact of the network papers within the published journal set was above average and the impact of the network papers in the fields was far above average. We further evaluated the global standing of the journal set containing the papers from the NIAID HIV/AIDS clinical trials networks relative to the fields in which the journal belong. The JCSm/FCSm ratio was 1.73, indicating the mean citation score of the network's journal set exceeded the mean citation score for all articles published in the fields to which the journals belong. Thus, the networks as a group publish in journals with a high impact in the fields of study with relevance to HIV.

**Table 1:** Performance and impact indicators for all NIAID HIV/AIDS clinical trials networks publications, 2006–2008.

Group	P	C	CPP	CPP/JCSm	CPP/FCSm	JCSm/FCSm
All network publications	419	2582	6.16	1.12	1.90	1.74
2008 publications	119	119	1.00	1.02	1.02	1.77
2007 publications	152	668	4.39	1.07	1.95	1.54
2006 publications	148	1795	12.13	1.15	2.03	1.77

Note: P = number of articles published; C = total number of citations; CPP = average number of citations per publication; JCSm = average expected citations for a journal set; FCSm = average expected citations across a combination of fields.

### Profile of interdisciplinary research

An important part of advanced bibliometric performance and impact evaluation is the construction and representation of a research profile for a specific entity [21], [23]. A research profile is a breakdown of output, performance, and impact according to internationally defined research fields on the basis of the journals used by the entity. In an effort to examine the variation in presence, performance, and impact of NIAID HIV/AIDS clinical trials networks papers in relation to a wide variety of scientific areas of study we conducted an analysis of bibliometric indicators by fields (i.e. ISI's Current Content Categories). Each paper (except letters, editorials, and meeting abstracts) is assigned a main field category label by Thomson-ISI and extracted from JCR. In our analysis, 29 of the 419 papers were not assigned a field category because of their type, resulting in a final set of 390 papers (general article and reviews) for the field analysis and profile generation.

The 390 NIAID HIV/AIDS clinical trials networks papers were distributed across 41 distinct field categories, reflecting a wide range of disciplines, although more than 36% (15) of the fields included only a single publication. Within these 41 fields, we first assessed the position of network papers as a means for understanding their presence, relative to the status of the journals within each field. To determine the status of each journal within each field, we rank-ordered the journals within each field by their Eigenfactor score [26]. The Eigenfactor score is a measure of the total influence of a journal based on cross-citation patterns. The Eigenfactor score provides several advantages to the JCR impact factor because it uses the 5 previous years for the target window and excludes self-citations. Thus, the ranking of a journal in a field, based on Eigenfactor score, is a robust indicator of the overall influence of the journal within the field [27]. Second, to determine performance of papers across fields, the total number of citations (*C*) and citations per paper (*CPP*) for the set of papers within each field was calculated. Finally, in terms of assessing impact of the papers across fields, we again calculated the field normalized measured impact ratios (*CPP/FCSm*) of NIAID HIV/AIDS clinical trials networks' publications for each field.

Overall, the scientific strength and international visibility of the NIAID HIV/AIDS clinical trials networks' publications across fields was high to very high. Nearly three-fourths (74.7%) of the 390 papers were published in the top quartile of ranked journals across fields. Furthermore, slightly more than one-third (35.2%) were published in the first, second, or third ranked journal in their respective fields. Table 2 displays the distribution of NIAID HIV/AIDS clinical trials networks' publications across fields, along with the respective positioning of publications within each field's journal hierarchy.

**Table 2:** Distribution of NIAID HIV/AIDS clinical trials networks' papers in journal hierarchy by field category (fields with 2 or more publications).

Field Category	N	Cites	Average cites per paper	# of papers in top quartile of ranked journals within field	# of papers in 1, 2, or 3 ranked journal within field
Infectious Diseases	169	1070	6.3	138	46
Pharmacology & Pharmacy	45	149	3.3	23	12
Medicine, General Internal	20	443	22.2	18	12
Virology	19	126	6.6	15	15
Microbiology	15	121	8.1	15	7
Public, Environmental & Occupational Health	12	32	2.7	5	4
Immunology	12	21	1.8	8	2
Clinical Neurology	9	96	10.7	7	6
Pediatrics	9	29	3.2	9	8
Medicine, Research & Experimental	8	157	19.6	1	1
Obstetrics and Gynecology	8	28	3.5	8	7
Statistics & Probability	6	20	3.3	4	0
Biomedical Research Methods	5	25	5.0	0	0
Medical Laboratory Technology	5	22	4.4	4	1
Chemistry, Analytical	4	25	6.3	2	0
Psychology, Multidisciplinary	4	11	2.8	4	0
Social Sciences, Biomedical	4	6	1.5	4	0
Biotechnology & Applied Microbiology	3	14	4.7	2	0
Healthcare Sciences & Services	3	8	2.7	2	1
Endocrinology & Metabolism	3	7	2.3	2	2
Biochemistry & Molecular Biology	2	8	4.0	1	0
Mathematical & Computational Biology	2	9	4.5	1	0
Nursing	2	2	1.0	1	1
Nutrition & Dietetics	2	0	0.0	2	2
Pathology	2	4	2.0	2	0
Veterinary Sciences	2	18	9.0	2	2
**Total**	**390**	**2516**	**6.5**	**289**	**135**

**Figure 1:**
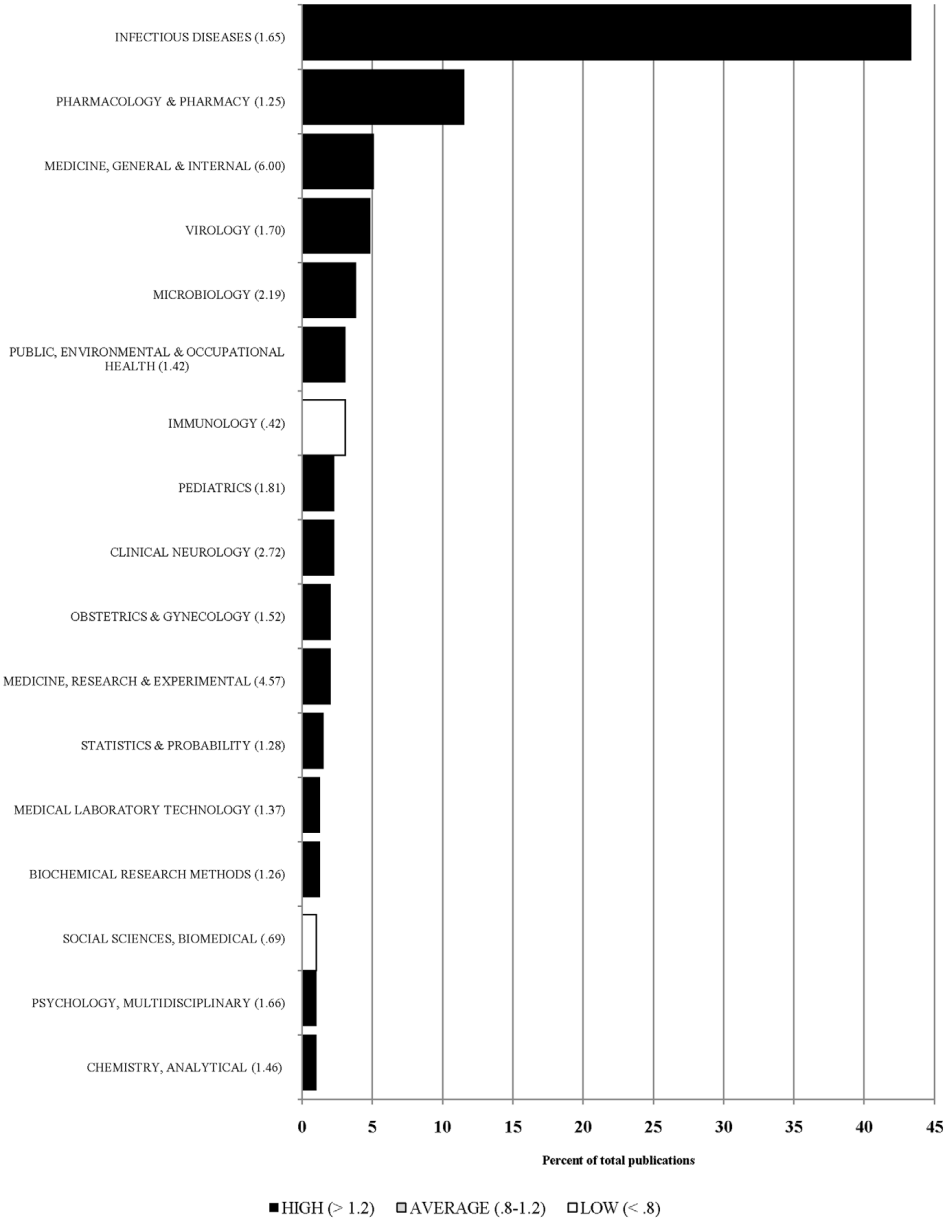
Research output profile for NIAID HIV/AIDS clinical trials networks, 2006–2008.

In terms of the impact assessment within each field, Infectious Diseases (*C* = 1070) had the largest number of citations, followed by Medicine, General and Internal (*C* = 443), reflecting the primary biomedical research focus the of NIAID HIV/AIDS clinical trials networks. The largest citations per paper values were found in the fields of Medicine, General and Internal (*CPP* = 22.15); Medicine, Research and Experimental (*CPP* = 19.63); and Clinical Neurology (*CPP* = 10.67) suggesting that NIAID HIV/AIDS clinical trials networks research is generating substantial interest in these disciplines. Figure 1 provides a spectral analysis of the research output of the NIAID HIV/AIDS clinical trials networks across those fields with more than a 1% share in the total number of publications. The color of the shaded bar corresponds to the average field normalized score for the set of papers published within the respective field. Results revealed that of the 17 fields with more than 1% of the total share of publications, the largest impact (*CPP/FCSm* above 2.5) was seen in the fields of Medicine, General and Internal; Medicine, Research and Experimental; and Clinical Neurology. A lower, but still substantial impact level (*CPP/FCSm* above 1.5) was also observed and included the fields of Microbiology; Pediatrics; Virology; Psychology, Multidisciplinary; Infectious Diseases; and Obstetrics and Gynecology. The field normalized citation scores indicated that publications in these fields were highly influential and visible, far exceeding the number of citations expected for publications of the same age and type in the respective fields. Even in those fields where relatively fewer papers were published, the measured impact of those papers was still high.

### Identification and impact of top papers

While the overall impact of the entire set of network papers was found to be slightly above the international averages for both journal and fields, we were also interested in identifying a subset of publications that might be making a substantive impact across HIV research environments. To that end, first we isolated a collection of papers that were determined to have the necessary number of citations needed to make a publication one of the 10% most cited publications of the same age, type, and within the same field. The number of papers within the top 10% of the worldwide impact distribution has been considered a robust indicator of scientific excellence in previous bibliometric studies [23], [28], [29]. At the 10% highly-cited threshold, 72 network papers were found, representing 18.5% of the total network publications. It was important to distinguish the nature of each of these papers, as it was anticipated that citation patterns vary as a function of the type of publication across this very interdisciplinary environment. Therefore, we categorized each of the 72 papers into one of six types, in order to help understand the differences in use and acceptance across fields. Papers were classified as follows: Primary publications - the ‘main’ publication from a research protocol reporting on the primary objectives; Secondary publications - additional protocol publications reporting results on secondary and/or tertiary endpoints; Review articles, Cross-protocol analyses, External collaborative research (typically with investigators from outside of a network), Observational studies, and Other (e.g. non-network supported study where network investigators were included as authors). For the categorization, each publication was independently reviewed by two authors (JK and JS) and consensus reached with a third (SR).

The results of the categorization of the 72 highly-cited papers revealed that nearly one third (31%) were publications detailing the primary results of a specific study protocol. Not surprising, nearly all of the primary study results publications were in the field of Infectious Diseases. Twenty-four percent were collaborative studies with groups outside of the networks, 18% were publications focused of results of secondary analyses, 12% were reviews, 6% were observational studies, 5% were cross protocol studies, and 4% were classified as other. This set of 72 papers was published in 26 different journals and acquired a total of 1,522 citations. They averaged 21.14 citations per paper with a median of 14.5. More than half (56.9%) were published in the core HIV-related fields of Infectious Diseases, Microbiology, and Virology. The average journal impact factor was 10.34, and values ranged from 1.71 to 52.59. In addition, 61.4% of the papers were published in the first, second, or third ranked journal within their respective fields, based on the ranking of journals by Eigenfactor scores. The performance and impact indicators for the 72 papers above the 10% highly cited threshold by category are listed in Table 3.

**Table 3:** Performance and Impact indicators for the 72 papers at the 10% highly-cited level by publication type.

Type	P	C	CPP	CPP/JCSm	CPP/FCSm	JCSm/FCSm
Total	72	1522	21.14	1.74	5.42	3.11
Primary study	22	528	24.0	1.66	6.62	4.02
Secondary study	13	216	16.62	3.38	5.77	1.75
Review article	9	219	24.33	1.05	4.90	4.49
Cross-protocol study	4	72	18.0	.84	3.27	3.93
Collaborative/external study	17	419	24.65	2.48	5.17	2.12
Observational study	4	18	4.50	1.95	4.69	2.08
Other	3	50	16.67	2.29	3.95	1.81

Note: P = number of articles published; C = total number of citations; CPP = average number of citations per publication; JCSm = average expected citations for a journal set; FCSm = average expected citations across a combination of fields.

To assess the scientific impact that these upper-tier publications might be generating, we again used the journal normalized citation score and the field normalized citation score. Collectively, the results revealed this set of 72 papers were performing at a very high level and far exceeded the average citations for papers of the same type, age, and within the same journal. The citations per paper (*CPP*) across all categories were high. More specifically, the papers publishing primary study results, secondary analyses results, and collaborative study results were found to far exceed the expected number of citations when compared to papers in the same journals and fields. This highlighted their widespread presence, performance and impact across global HIV research environments and confirmed their highly-cited threshold status and influence relative to similar papers in the journals in which they were published.

### Co-authorship and collaboration patterns

Analysis of co-authorship patterns is frequently used in advanced bibliometric studies as a means for understanding collaboration. In assessing the degree of collaboration across the NIAID HIV/AIDS clinical trials networks' studies, we examined the co-authorship patterns. A total of 2,834 authors were listed across the 419 papers. We found 11 authors on average for each paper submitted by the NIAID HIV/AIDS clinical trials networks, indicating a high degree of co-authorship. The wide range of authors was a factor in the breadth of scientific specialties, resulting in a relatively low disciplinarity score for these publications. Specifically, a disciplinarity score is a measure that reflects the level of multidisciplinarity in a set of papers. Often referred to as the Herfindal index, this metric range is from 0–1 and equals the sum of squares of disciplinary shares for the set. The lower the number is, the more multidisciplinary the group of papers [30]. In the full set, we found the network papers reflect a fairly wide range of disciplines due to its relatively low disciplinarity score (0.14). Furthermore, the degree of international collaboration based on co-author relationships was considerable, with US-based authors collaborating with authors in 41 different countries on a total of 243 papers. As the largest HIV/AIDS clinical trials research system in the world, this finding was not surprising and confirmed the extensive involvement of international investigators and their contributions to the clinical trials research networks.

**Figure 2:**
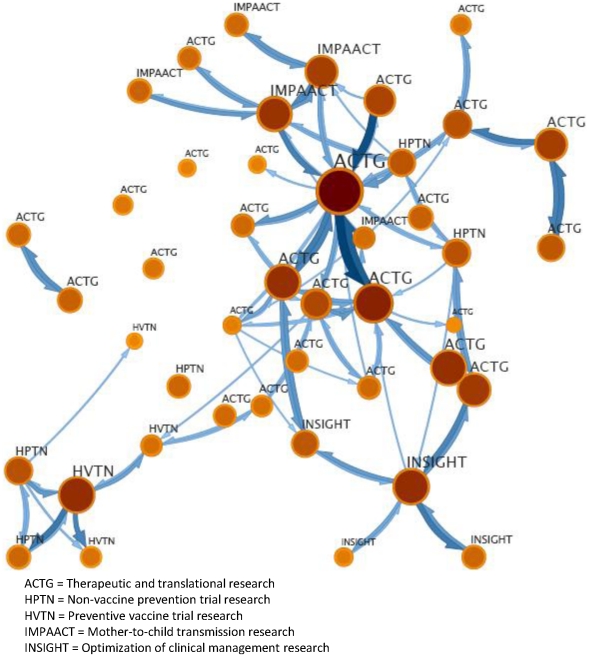
A 44-cluster co-authorship network of papers at the 10% highly-cited threshold.

In an effort to visually model the interdisciplinary of the NIAID HIV/AIDS clinical trials networks-funded research, we first created a co-authorship network for the 72 highly-cited articles. Disambiguation of author names is a central issue in collaboration network analysis and strategies range from complex algorithms to manual cleaning depending on the size of the data set and purpose of the analysis [31]. Given our purpose to model a co-authorship network using a small subset of papers, we manually reviewed and verified each of the 72 highly cited network papers in the WoS database to identify any ambiguities. Upon the validating the correct full bibliographic record paper in the WoS, we used the author's initials and institutional affiliation to clarify any multiple occurrences of similar names to ensure an accurate match of authors. This cleaned list of highly cited papers was downloaded in a field tagged format and a co-authorship analysis was conducted using CiteSpace software developed for network analysis and visualization based on publication data [32]. We then subjected the network output to a clustering method specifically designed to represent local interactions among subunits of a larger system using links and nodes in directed, weighted networks [33]. In modeling the intra-network connectivity of highly-cited papers, we sought visual simplicity and the most parsimonious representation of a complex set of collaborative relationships revealed in the co-author pairings. Figure 2 displays the co-authorship network for the most productive author relationship clusters or modules for the set of 72 papers above the 10% highly-citied threshold.

Each module includes a group of nodes (i.e. authors), which are aggregated into a single well-connected module. The original 634 nodes from the network analysis output were clustered into 44 modules, connected by 103 inter-module links. Links between modules represent pair-wise relationships where the more heavily weighted links (i.e. thicker lines) indicated greater co-author pairing between modules. As shown in Figure 2, every module represents a cluster of nodes and the links between the modules represent the flow between the modules, in this case the co-authorship pairings. Each module was then labeled to represent the network from which the aggregated set of authors was affiliated, providing a coarse grained view of cross-network collaboration on the highly-cited papers.

Through the examination of the extensive co-authorship network, we observed the emergence of co-authorship patterns of the highest performing papers over the three year period across the entire NIAID HIV/AIDS clinical trials system. Overlaying the network specific areas of research illuminates the interconnectivity and collaboration between researchers from different parts of the system. The prominence of ACTG-supported research was evident in the model. This network has the longest history and largest infrastructure for conducting HIV/AIDS clinical trials research. As such, ACTG submitted the most publications and had the largest number of papers in the highly-cited group. While larger module size corresponds to the relative significance in the network, several important connections between smaller collaborative groups who function as bridges to different, but related research communities were also found. For example, in the lower left hand corner of the network map is a group focused on preventive vaccine trial research (supported by HVTN). Connected to this group of researchers by virtue of their shared author teams, are multiple groups of scientists focused on non-vaccine prevention trial research (supported by HPTN). Similarly, we found several instances where author teams focused on both preventive non-vaccine and preventive vaccine trial research were connected to therapeutic and translational research (supported by ACTG) research groups. The connections observed between these research teams suggest collaborative interactions across research foci and reflects the integration of prevention and treatment research – one of the primary goals in restructuring the networks. Overall, the visualization of co-authorship patterns at the highest level of publication productivity revealed greater cross-network collaboration than what could be extracted from a general review of author teams and affiliations found in the bibliographic record.

### Inclusion of papers in research syntheses

Unfortunately the gap between clinical research findings and clinical practice implementation in many areas of health care and public health is large, well documented, and disconcerting [34]. Assessing the progression of scientific output, as initial research findings move on their way to becoming improvements in clinical practice, provides some insight into the translation of publicly funded research development from new basic insights into use in the public's health care. As clinical research is disseminated through the peer-reviewed literature to others in the field it becomes available for inclusion by those who are responsible for conducting research syntheses and/or developing guidelines to aid in determining clinical practice. Eventually these syntheses and guidelines are practically applied by medical staff and health program developers when implementing or adjusting healthcare programs to accommodate the advancements in therapies and other treatments. Following the simple, but logical path of awareness in the literature, acceptance in the synthesized research, and adoption into practice [35] we sought to determine the extent to which network publications were cited in synthesized reviews.

We found 187 papers from the set of 419 publications from the NIAID HIV/AIDS clinical trials networks were cited by 365 review articles published between 2006 and 2009. As categorized by JCR, citing reviews can encompass a range of syntheses, including reviews of the literature, clinical practice guidelines, and meta-analyses. The 187 network papers found in these reviews were top performing publications with 63 (33.7%) found to be above the 10% highly-cited threshold. More than half of the 187 papers (52.4%) were published in 2006, 36.9% in 2007, and 10.7% in 2008. In terms of the type of reviews citing network papers, 348 of the 365 were classified as general literature reviews of the research. In addition, we found a small set of specialized research syntheses. Within this subset of research syntheses were 11 practice guidelines (directions or principles to assist the health care practitioner with patient care decisions about appropriate diagnostic, therapeutic, or other clinical procedures for specific clinical circumstances), 5 meta-analyses (studies using a quantitative method of combining the results of independent studies that address a set of related research hypotheses), and 1 general guideline (statements, directions, or principles presenting current or future rules or policy focused on general conduct and administration of health care activities). The number of NIAID HIV/AIDS clinical trials network research papers cited in the 348 research literature reviews was 159, and 28 network papers were cited in the 17 specialized research syntheses. In order to determine the performance and impact of the NIAID HIV/AIDS clinical trials network publications included in these reviews and syntheses, citation data was aggregated and analyzed according to type using the same metrics used previously in this report. The results are represented in Table 4 and confirm the wide recognition and use of these publications across journals and fields of relevance to HIV research. These results suggest that a large proportion of network produced papers was of special interest to those synthesizing the current research, and were performing well above average and influential across HIV-related fields.

**Table 4:** Performance and impact indicators for NIAID HIV/AIDS clinical trials networks publications cited in research reviews and syntheses, 2006–2009.

Set	P	C	CPP	CPP/JCSm	CPP/FCSm	JCSm/FCSm
Research literature review	159	1503	9.45	1.23	2.19	1.80
Practice guidelines	18	514	28.56	1.44	5.79	4.01
General guidelines	5	79	15.80	3.70	5.31	1.44
Meta-analyses	5	94	18.80	2.37	3.70	1.56

Note: P = number of articles published; C = total number of citations; CPP = average number of citations per publication; JCSm = average expected citations for a journal set; FCSm = average expected citations across a combination of fields.

The 365 citing reviews were published in 196 different journals, and to date had received a total 1752 cites with an average of 5 cites per paper. These reviews were distributed across 52 different fields, with the greatest number of reviews and syntheses found in Pharmacology and Pharmacy (98), Infectious Disease (91), and Immunology (88). Using a number of different PubMed search filters, we identified major research support categories for the set of citing reviews, providing a sense of the primary funding support for the reviews that included papers from the NIAID HIV/AIDS clinical trials networks. Slightly more than one-third of the review articles were identified as non-US Government supported research. Research reviews and syntheses in this category are supported by domestic societies, institutes, state governments, universities, private organizations, as well as by foreign governments, academic institutions and private organizations. Moreover, one-quarter of the research reviews were supported by NIH sources, both intramural and extramural, and another quarter were supported by the federal Public Health Service (PHS), its bureaus, and services.

Collectively, these findings suggest that, despite the very short publication life of the papers from the NIAID HIV/AIDS clinical trials networks, their uptake in the synthesized research base had already been initiated. Previous research found an 8-year lag-time between publication of a cited paper and the publication of guidelines in the UK, suggesting a substantial delay in the appearance of research findings in the practice-based literature [36]. The rapid inclusion of papers from the DAIDS networks in guidelines and meta-analyses is notable; as it is expected that acceptance of the research output in reviews and syntheses will increase across the entire set of papers over time.

## Discussion

This study used advanced bibliometric methods to evaluate the presence, performance, and impact of peer-reviewed publications from the NIAID HIV/AIDS clinical trials networks from 2006–2008. Through this approach, we sought to expand traditional bibliometric analyses beyond citation counts to include normative comparisons, network analysis and visualization techniques, and assessment of inclusion in reviews and syntheses. Expanding descriptive and inferential bibliometric methods are especially important in new research frontiers like prevention [37], as well as in very collaborative and cross-disciplinary environments. Overall, the scientific publications from the NIAID HIV/AIDS clinical trials networks were slightly above the average number of citations worldwide controlling for the journals in which they were published. Papers from the networks were published in journals that represented core areas of AIDS research, and for the most part, publications were found in highly recognized journals suggesting that network research is being disseminated to a large audience across AIDS-related fields. The daily practice of scientific research shows that scientists generating high quality research generally aspire to publish in the best journals, especially in the natural, biomedical, and medical sciences [23]. The presence of publications from the NIAID HIV/AIDS clinical trials networks at elevated levels of the journal hierarchy confirmed the scientific output was of high quality and researchers' work was meeting rigorous peer review standards for publication.

Self-citation rates were not unusual and did not contribute to greater than expected citation levels. While the set of papers from the NIAID HIV/AIDS clinical trials networks reached the expected level of performance, substantive impact was associated with a set of highly-cited publications. This collection of papers received significantly greater number of citations than what was expected, suggesting these papers were widely recognized by other scientists across the AIDS research environments. Moreover, the multidisciplinary nature of the NIAID HIV/AIDS clinical trials networks was evident in the high number of average authors per paper, the variety of journals, and the breadth of fields represented by the journals. The extensive global network of collaborative scientists was apparent through the international representation on author teams as well as the visual linkages found between highly productive author groups functioning as information brokers.

Research from the NIAID HIV/AIDS clinical trials networks was represented in published guidelines, both policy and procedural as well as clinical practice, highlighting the value of a subset of research output in shaping healthcare service delivery. The inclusion of NIH-sponsored clinical trials research, in reviews supported by non-US government sources (domestic and international) signals a broad acknowledgement and acceptance of the scientific output across a diverse scientific infrastructure. As a world leader in AIDS research, it is important that evaluation and monitoring of scientific output from the research networks include the translation of publicly-funded clinical research into clinical practice. Most clinicians' knowledge of published research is incomplete due to poor presentation research findings [38], lack of time to search for information, findings dispersed across large number journals [39], and difficulty in interpreting published evidence [38]. Reviews often address these problems by acting as the aggregators of current clinical research and serve as a source for evaluating the use of scientific results.

### Implications

Bibliometric assessment of research presence, performance, and impact has been based on a core assumption that scientists with important information actively seek to publish their findings in open, international journals [23]. Research scientists within the NIAID HIV/AIDS clinical trials networks are no different. With the focus of the networks on clinical trials research for HIV prevention and treatment, the dissemination and use of results within the professional literature is a widely accepted aspect of documenting the scientific accomplishments.

The results of this advanced bibliometric analysis have several important implications for evaluation and monitoring of the scientific output of the NIAID HIV/AIDS clinical trials networks and potentially other clinical trials consortia. First, this bibliometric study revealed key data collection and retrieval considerations. Going forward, specific guidance for recording and verifying bibliographic records will be critical to ensuring an appropriate match between network publications and citation data extracted from multiple bibliometric databases. Furthermore, as the multidisciplinary nature of the network publications continues to expand, important bibliometric data may need to be accessed and verified from other citation tracking databases, such as PubMed, Google Scholar and Scopus in addition to those found in WoS. Second, the variety of disciplinary fields found to contain network publications revealed variable expectations in terms of relative performance and impact. Thus, ongoing evaluation and monitoring of publication presence, performance, and impact should include analysis by field, with comparisons made to worldwide citation and publication measures. Normalization of citation and journal influence data from the international set of journals and fields is required for accurate assessment and comparison to determine the levels of presence, performance, and impact. Third, the results of the bibliometric study provide a baseline level of presence, performance, and impact, and suggest a set of potential indicators that may be applied to future analysis of output from the networks. For example, measures such as the “percent of network papers published in the top quartile of the respective fields” or the “percent of papers at the 10% threshold” may be employed in the future as indicators of enterprise research performance. Since papers at the 10% highly cited threshold were highly influential within the journals and fields in which they were published, setting expectations for increasing the number of papers reaching the threshold would quickly indicate the progress the networks were making toward meeting the objective of producing impactful research. Finally, in this study we examined the use of unique bibliometric techniques that, with further refinement, may provide a more robust picture of performance and impact beyond traditional citation analyses. Specifically, the examination of the presence of network papers in research syntheses extends the understanding of how and in what ways the scientific research produced by the networks is being utilized. The use of clinical guidelines as an intermediate indicator of research utility in the translational process [36] can provide a clearer picture of the downstream impact network research may be generating in terms of stimulating new research, supporting and confirming existing knowledge, or shaping clinical practices.

Moreover, while visualization techniques like those employed here may appear to be somewhat limited as a tool for evaluating performance or impact, they have utility as a heuristic for monitoring author or subject collaboration and identifying cross-network research efforts associated with high performance and impact. Indeed, similar network assessments have confirmed the widespread belief that many research frontiers are being driven by cross-fertilization of ideas, interdisciplinary collaborations, and further integration of scientific disciplines [40]. Moreover, network analysis and visualization techniques can be used to monitor strategic goals such as integration and collaboration across research areas over time. New methods are emerging that enable a more precise assessment of co-authorship impact on scientific publications and are particularly useful in interdisciplinary research environments where contributions are widely distributed [41]. Advanced bibliometric techniques, integrated systematically with other methods for assessing the full range of impact of scientific output, can yield important information for evaluation and monitoring an entity's productivity.

### Limitations

Although bibliometric methods have expanded across the research evaluation landscape issues related to coverage, communication practices among scholars and language have been noted [42]. For example, bibliometric analyses can only be applied to the published literature in journals that are indexed with respect to citations and do not cover unpublished works, works in non-indexed journals, and non-journal printed works such as books, dissertations, reports, or government documents. Citations are also treated as equal regardless of whether a work is being cited for its positive contribution to a field or being criticized as for its negative impact or poor quality. Different authors employ differing levels of care in compiling references and differences in citation tracking across databases remains a concern. The determination of impact through the exclusive use of bibliometric measures has received widespread criticism from the scientific community. Plenty has been written on the deleterious effects of the misuse of bibliometric data in judging rank, output, and value [43]–[45]. Caution must be employed in emphasizing the scientific quality of the research output *solely* on the basis of bibliometric data.

As with any statistical endeavor, bibliometric analyses have the potential to generate misleading and biased results. Because citation counts are time, type and field dependent, adjustment to the expected patterns for citations must be taken into account when comparing performance and impact. Although it is common for clinical trials papers to have large author teams, the average number of authors across papers was high. A large set of diverse authors on papers may increase the likelihood of an elevated number of citations and thus affect the assessment of performance. In future studies, it might be important to control for the number of authors to avoid inflated citation counts and more precise estimates of performance and impact. In addition, given the brief assessment period, a large proportion of the papers did not have an opportunity to establish their presence in the publication domain. Therefore, our analysis may not have captured the full variability of citations patterns of a completely mature set of publications. Nonetheless, where possible, we sought to isolate publication groups and normalize citation data to more precisely determine relative impact. Standard bibliometric techniques are well suited for assessing the contribution to the advancement of knowledge, but much less so in assessing contributions to mission-oriented objectives of research institutions, such as impacts to population health or collaboration among scientists. In addition, we attempted to examine some of the initial translational patterns in scientific output using bibliometric methods. However, our ability to do so was fairly limited, compounded by well-known issues in determining downstream effects of research outputs and the integration of different citation databases. Thus, these results are merely suggestive of the movement of the clinical research output from awareness to acceptance.

### Conclusion

Despite the limitations, advanced evaluative bibliometric analyses such as those conducted here, can be used to explicate and describe patterns of performance and impact of scientific research across a multidisciplinary research enterprise. At the management and policy level, bibliometric analysis has been identified as one of the tools that have potential to assist decision-makers in understanding science and innovation, investing in science and innovation, and using the “science of science” policy to address national priorities [46], [47]. Any publicly funded research enterprise must be held accountable for its productivity and advanced bibliometric analyses offer a set of sophisticated tools that can provide important evidence during evaluation. As a means for assessing scientific output, bibliometrics can help create a data-driven picture of scientific research within the publication landscape and offer evidence-based descriptions, comparisons, and visualizations of research output.
